# Early survival factor deprivation in the olfactory epithelium enhances activity-driven survival

**DOI:** 10.3389/fncel.2013.00271

**Published:** 2013-12-24

**Authors:** Adrien François, Iman Laziz, Stéphanie Rimbaud, Denise Grebert, Didier Durieux, Edith Pajot-Augy, Nicolas Meunier

**Affiliations:** ^1^INRA, UR1197 Neurobiologie de l'Olfaction et Modélisation en ImagerieJouy-en-Josas, France; ^2^IFR144, NeuroSud ParisGif-Sur-Yvette, France; ^3^Université de Versailles Saint-Quentin en YvelinesVersailles, France

**Keywords:** Olfaction, activity-dependent plasticity, EOG, U131, WISH

## Abstract

The neuronal olfactory epithelium undergoes permanent renewal because of environmental aggression. This renewal is partly regulated by factors modulating the level of neuronal apoptosis. Among them, we had previously characterized endothelin as neuroprotective. In this study, we explored the effect of cell survival factor deprivation in the olfactory epithelium by intranasal delivery of endothelin receptors antagonists to rat pups. This treatment induced an overall increase of apoptosis in the olfactory epithelium. The responses to odorants recorded by electroolfactogram were decreased in treated animal, a result consistent with a loss of olfactory sensory neurons (OSNs). However, the treated animal performed better in an olfactory orientation test based on maternal odor compared to non-treated littermates. This improved performance could be due to activity-dependent neuronal survival of OSNs in the context of increased apoptosis level. In order to demonstrate it, we odorized pups with octanal, a known ligand for the rI7 olfactory receptor (Olr226). We quantified the number of OSN expressing rI7 by RT-qPCR and whole mount *in situ* hybridization. While this number was reduced by the survival factor removal treatment, this reduction was abolished by the presence of its ligand. This improved survival was optimal for low concentration of odorant and was specific for rI7-expressing OSNs. Meanwhile, the number of rI7-expressing OSNs was not affected by the odorization in non-treated littermates; showing that the activity-dependant survival of OSNs did not affect the OSN population during the 10 days of odorization in control conditions. Overall, our study shows that when apoptosis is promoted in the olfactory mucosa, the activity-dependent neuronal plasticity allows faster tuning of the olfactory sensory neuron population toward detection of environmental odorants.

## Introduction

Among all sensory systems, only olfaction can be modulated at the peripheral level by neuronal survival as only olfactory sensory neurons (OSNs) are renewed throughout the lifetime of the animal (Graziadei, 1973). Indeed, as OSNs are in direct contact with the environment, they are under permanent aggression from oxidative stress, pathogens or xenobiotics and thus have a limited life expectancy. They regularly undergo apoptosis and are renewed from basal cells (Schwob, 2002). It has been clearly demonstrated that the olfactory epithelium (OE) expresses anti-apoptotic factors affecting OSNs (Moon et al., 2009) but the existence of activity-dependant survival of OSNs has remained controversial. The modification of brain circuitry by neuronal activity has been widely studied since the first demonstration of its importance in the formation of ocular dominance columns (Wiesel and Hubel, 1963) or the survival of motor neurons during chick embryo development (Hollyday et al., 1977). Several studies have since demonstrated that some of these modifications rely on the secretion of trophic factors such as BDNF by or around the most active neurons (Castren et al., 1992; Jones et al., 2007). The first evidences of such survival signals in OSNs arise from unilateral naris occlusion studies (Farbman et al., 1988). Although informative on activity-driven survival mechanisms of OSNs, such studies are controversial as they combine a decrease of odor stimulation with other effects including asymmetry of environment toxicity (Coppola, 2012). However, it clearly shows that the environment modulates OSN life span with olfactory receptor (OR) specificity (Rimbault et al., 2009; He et al., 2012; Zhao et al., 2013). Other studies have directly focused on modulating the odorant content of the environment. Most pointed out that the presence of odorant in the animal environment increases the population of OSNs sensitive to this specific odorant (Wang et al., 1993; Watt et al., 2004; Jones et al., 2008) but other studies did not show any change (Kerr and Belluscio, 2006) if not a decrease (Cavallin et al., 2010). The controversy in literature dealing with activity dependent OSN survival thus remains to be addressed.

We had previously shown that endothelin acts as an anti-apoptotic factor on olfactory epithelial cells (Laziz et al., 2011). In order to explore the effect of anti-apoptotic factor impairment in the olfactory epithelium, we performed intranasal delivery of endothelin receptor antagonists in the OE of newborn rats. Such treatment led to an increase of apoptosis level in the OE and a decrease of OSN responses amplitude recorded by electroolfactogram (EOG). Despite these changes in OSN population, the treated animals performed better than their control littermates in an orientation test based on maternal odors detection. We hypothesized that the paradoxical increase in olfactory performances that we observed while depriving the olfactory mucosa of endothelin may be caused by an increased survival of OSNs activated by an odorant already present in the environment. In a pro-apoptotic context, such survival would alter the population toward more OSNs sensitive to odorants present in the environment. As specific OSNs sensitive to maternal odorant cues are unknown, we odorized the pup's mother with various concentrations of octanal, known to activate the rI7 olfactory receptor (Zhao et al., 1998) and we quantified the rI7-expressing OSN population. Our results suggest that, unlike other systems where neuronal plasticity is strongly dependent on activity (e.g., visual and motor systems), the olfactory epithelium exhibits moderate activity-dependent neuronal survival, except under pro-apoptotic conditions. When apoptosis is promoted, such neuronal plasticity is enhanced, allowing faster tuning of the OSN population toward detection of odorants present in the environment. The controversy of the literature focused with activity-dependent OSN survival may thus arise from its moderate impact on cellular dynamics in a physiological context.

## Results

### Intranasal treatment with endothelin receptor antagonists increased apoptosis in the OE and reduced the OSN responses to odorants

**Figure 1:**
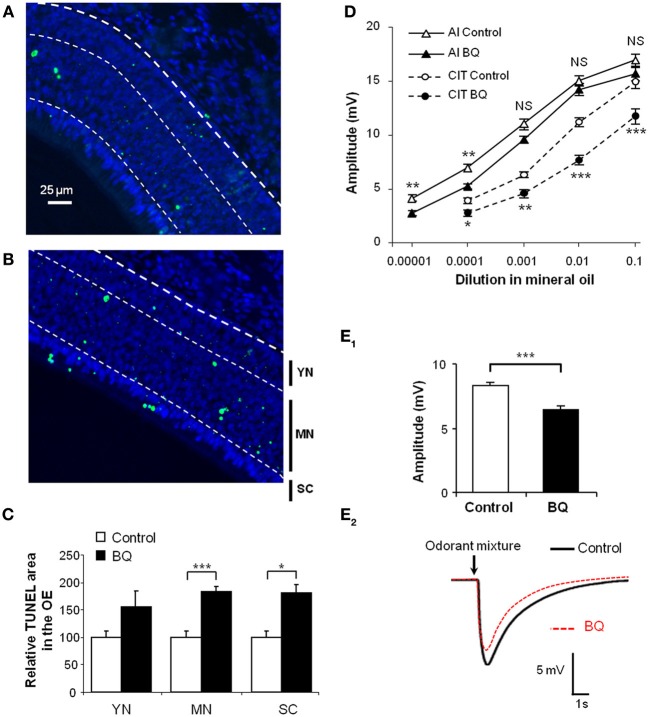
**Increase of apoptosis level in the OE and decrease of EOG responses after BQ treatment. (A,B)** Representative images of TUNEL assay in the OE from 10 days old pups treated with vehicle **(A)** or BQ **(B)**. The bold dotted white line represents the basal lamina, used to delimit the area of the OE; the thin dotted white lines represent the limits of the different cell layers corresponding to basal cells and immature neurons (YN for young neurons); mature neurons (MN) and sustentacular cells (SC). The level of apoptosis was quantified as a percentage of area with TUNEL staining in each cell layer taken separately **(C)**. Results were expressed as the mean of TUNEL signal normalized to vehicle treated littermates ±s.e.m. (*n* = 10; ^*^P < 0.05; ^***^P < 0.001). **(D,E)** EOG responses to various odorants in animals treated with BQ or vehicle. Values represent the mean of peak amplitudes (±s.e.m.) values (*n* = 12; ^*^P < 0.05; ^**^P < 0.01; ^***^P < 0.001; control vs. BQ treated). Odorant stimulations were performed with increasing concentrations of isoamyl acetate (AI) and citral (CIT) **(D)**, or a mixture of 12 odorants at 10^−3^M **(E_1_)**. Representative recordings of responses to 200 ms stimulus of the odor mixture **(E_2_)**.

We had previously shown that endothelin acts as an anti-apoptotic factor for olfactory epithelium cells *in vitro* (Laziz et al., 2011). In order to impair the olfactory epithelium of endothelin induced survival signal, we treated pups by an intranasal mixture of endothelin receptors ET_*A*_ and ET_*B*_ antagonists, respectively, BQ_123_ and BQ_788_, both at 10^−5^M. For simplicity, this mixture will be referred as BQ. The treatment was performed from P1 to P10 twice a day. We evaluated the level of apoptosis by TUNEL quantification between control (vehicle) and treated pups (BQ) in the dorso-medial area of the OE. We chose this zone since it displays relatively stable levels of neuron survival in wild type animals (Vedin et al., 2009). We further delimited three sub-areas of the OE in order to separately assess the TUNEL-stained nuclei originating from young neurons and basal cells (YN; 25% area of OE above lamina propria), mature neurons (MN; 50% middle area of OE) and sustentacular cells (SC; 25% apical area of OE) (Moon et al., 2009; Laziz et al., 2011). Compared to control littermates, the level of apoptosis was statistically increased in the mature neurons and sustentacular cells layers of the olfactory epithelium from treated animals (183.4 ± 8.8 vs. 100 ± 10.5% and 180.7 ± 11.3 vs. 100 ± 10.8% respectively; *n* = 12; Figures 1A–C).

To assess whether the increase of apoptosis in the OE could impact the OSN responses to odorants, we performed EOG recordings on turbinates IIb and III that are easily accessible and which give regular and stable responses in our hemi-head preparation. We stimulated the OE with increasing concentrations of isoamyl acetate and citral (Figure 1D) as well as a mixture of 12 odorants diluted in mineral oil (Figure 1E). We measured the maximum amplitude of responses to odorant for control and treated animals. The responses to odorants were globally decreased for animals treated with BQ. This decrease was significant except for the three highest concentrations of isoamyl acetate and the strongest effects were observed for the stimulations with citral at 1:100 and the odorants mixture (11.1 ± 0.5 vs. 7.6 ± 0.6 mV and 8.3 ± 0.3 vs. 6.4 ± 0.3 mV, respectively; *n* = 12).

### Treated animals performed better in an odor based orientation test

**Figure 2:**
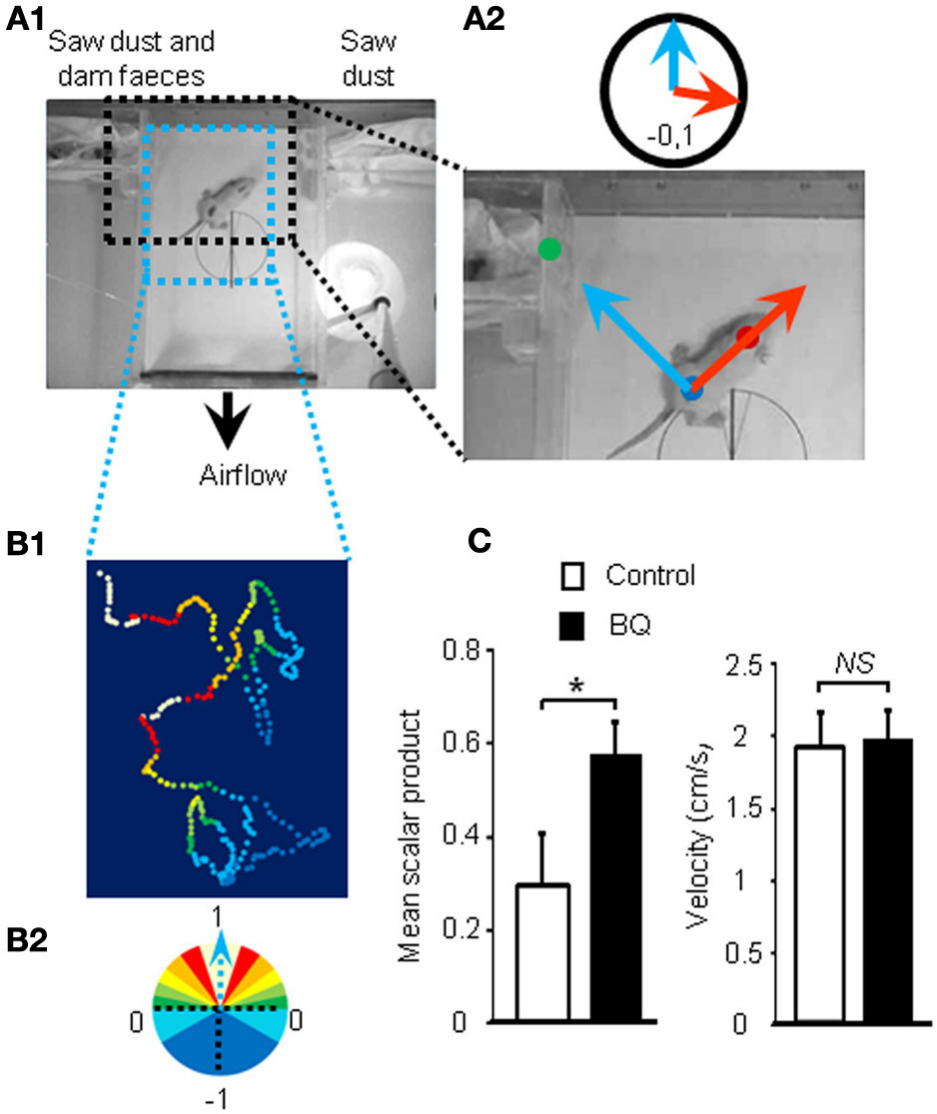
**Improvement of olfactory performances after 10 days of endothelin receptor antagonists treatment. (A_1_)** Pups were placed in the center of a box with a continuous flow of incoming air from two different compartments. **(A_2_)** Scalar products of two vectors were calculated to score the orientation of the animal toward the dam odor source. **(B_1_)** Representative path and orientation of a pup's head position during a 1 min trial. Colored dots represent head position in the box and orientation toward the odor source, ranging from white to dark blue, from full orientation to opposite orientation toward dam odor source, i.e., 1 to −1 scalar product, respectively **(B_2_)**. **(C)** Mean scalar product and mean of velocity values (±s.e.m.) of pups treated with BQ or vehicle (*n* = 12; ^*^P < 0.05).

As the treatment with intranasal endothelin receptor antagonists led to an increase of apoptosis in the OE and a decrease in EOG amplitudes, we next investigated if such modulations could impact the animal's ability to detect odorants. Ten days old pups are still blind and thus rely mainly on olfactory cues for orientation in a two choices test. We designed an orientation test for pups based on maternal odor detection (Figure 2A_1_). We calculated the scalar product of two unit vectors to score the orientation of the pup during the test (Figure 2A_2_). The scalar product ranges from 1 to –1, indicating a direct or opposite orientation of the pup toward the maternal odor source, respectively. A typical tracking of one pup is displayed on Figure 2B and its associated movie is accessible in Additional file 1. Most pups were able to find the dam odor source as the mean scalar is above 0 but surprisingly the animals treated with endothelin receptor antagonists, i.e., displaying a higher level of apoptosis in the OE, performed significantly better (0.35 ± 0.11 vs. 0.57 ± 0.08 for control and treated pups, respectively; Figure 2C). The velocity was not statistically different between both groups (1.91 ± 0.25 vs. 1.96 ± 0.2 cm/s, respectively; Figure 2C).

### rI7-expressing OSN population is decreased by treatment in absence of rI7 ligand

**Figure 3:**
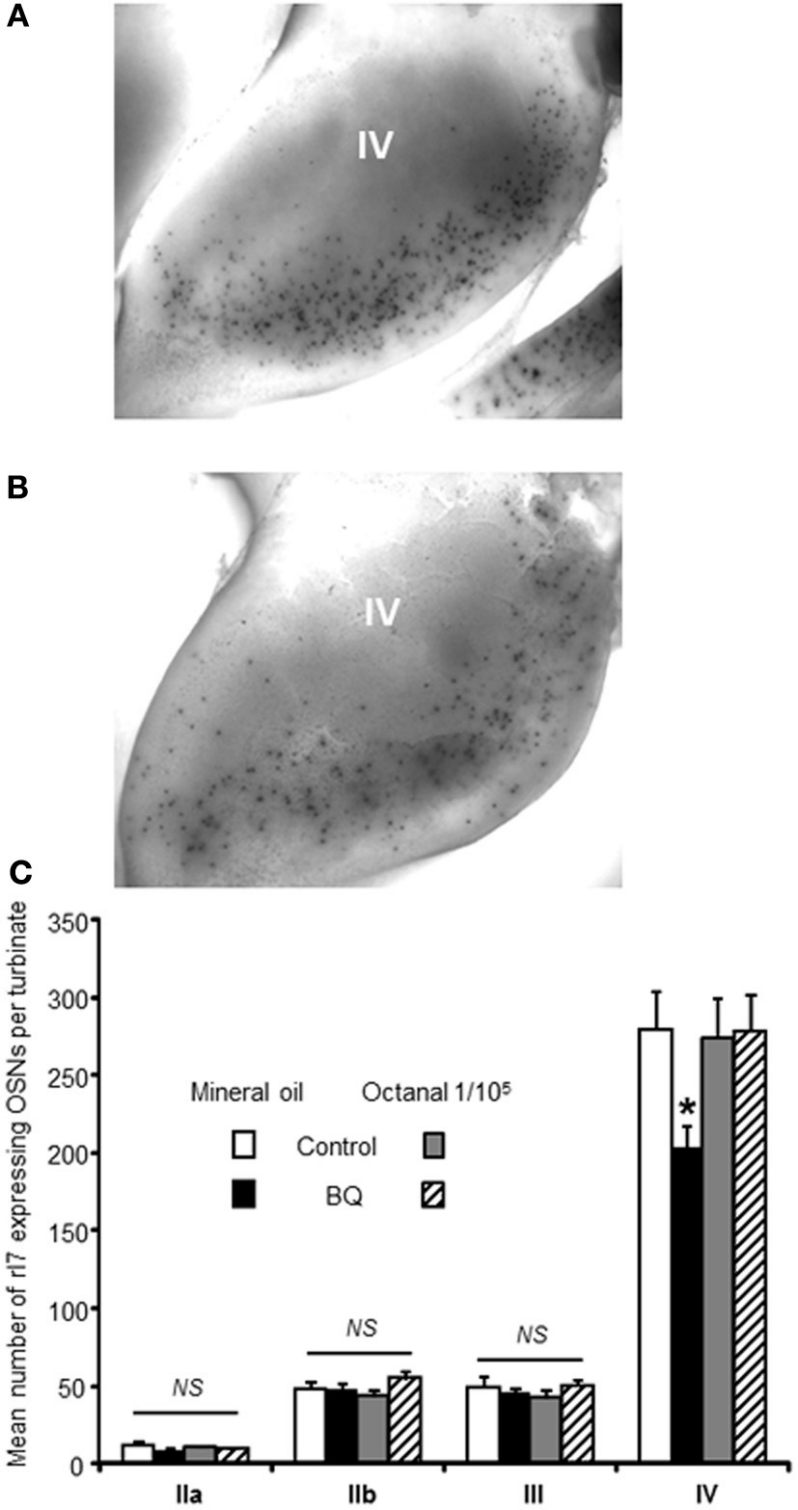
**Number of rI7-expressing OSNs is decreased by BQ except in presence of its cognate ligand octanal**. Representative picture of turbinate IV after whole mount *in situ* hybridization against rI7 for **(A)** a control animal or **(B)** a BQ treated animal both odorized with mineral oil. **(C)** The number of rI7-expressing OSNs present at the surface of the turbinates was compared between pups treated with endothelin receptor antagonists (BQ) or control littermates. Dam was sprayed with either mineral oil or octanal diluted in mineral oil during pup's treatment. Values are expressed as the mean number of rI7-expressing OSNs ± s.e.m. (*n* = 6; ^*^P < 0.05).

Recent studies have clearly pointed out that the OSN population could be modulated according to odorants present in the environment (Santoro and Dulac, 2012; Zhao et al., 2013). To explain our contradictory results, we hypothesized that while our treatment reduced globally the OSN population by increasing apoptosis, the OSN population of treated animal would contain a higher proportion of OSNs stimulated by environmental odorants because their survival would be promoted. Indeed, OSNs sensitive to pup's mother odorants would enter apoptosis less than non-stimulated OSNs, conferring a better sensitivity toward maternal odorants for treated animals. As mature OSNs express only one type of olfactory receptors (ORs), an OSN population responding to one odorant can be evaluated by following the expression of one OR in the OE (Khan et al., 2011). Unfortunately, the ORs sensitive to maternal odorants are unknown, preventing us from following this particular OSN population. To circumvent this difficulty, we focused our study on the rI7 OR, one of the best characterized OR in rats (Zhao et al., 1998). We first examined if our treatment led to a decrease of OSN population expressing rI7. We counted the number of rI7-expressing OSNs present at the surface of turbinates using whole mount *in situ* hybridization (WISH, Figures 3A,B). While the number of OSNs on turbinates IIa, IIb and II was stable between control and treated animal, it was significantly affected in turbinates IV (279 ± 23 vs. 203 ± 14 OSNs for control and treated pups, respectively; Figure 3C). To test our hypothesis of activity-driven survival of OSNs in our context, we odorized the dam with octanal along the intranasal delivery of endothelin receptor antagonists. Such odorization was enough to reverse the effect of increased apoptosis in the OE of treated animal (273 ± 26 vs. 277 ± 24 OSNs, respectively).

### Specificity of odorization

**Figure 4:**
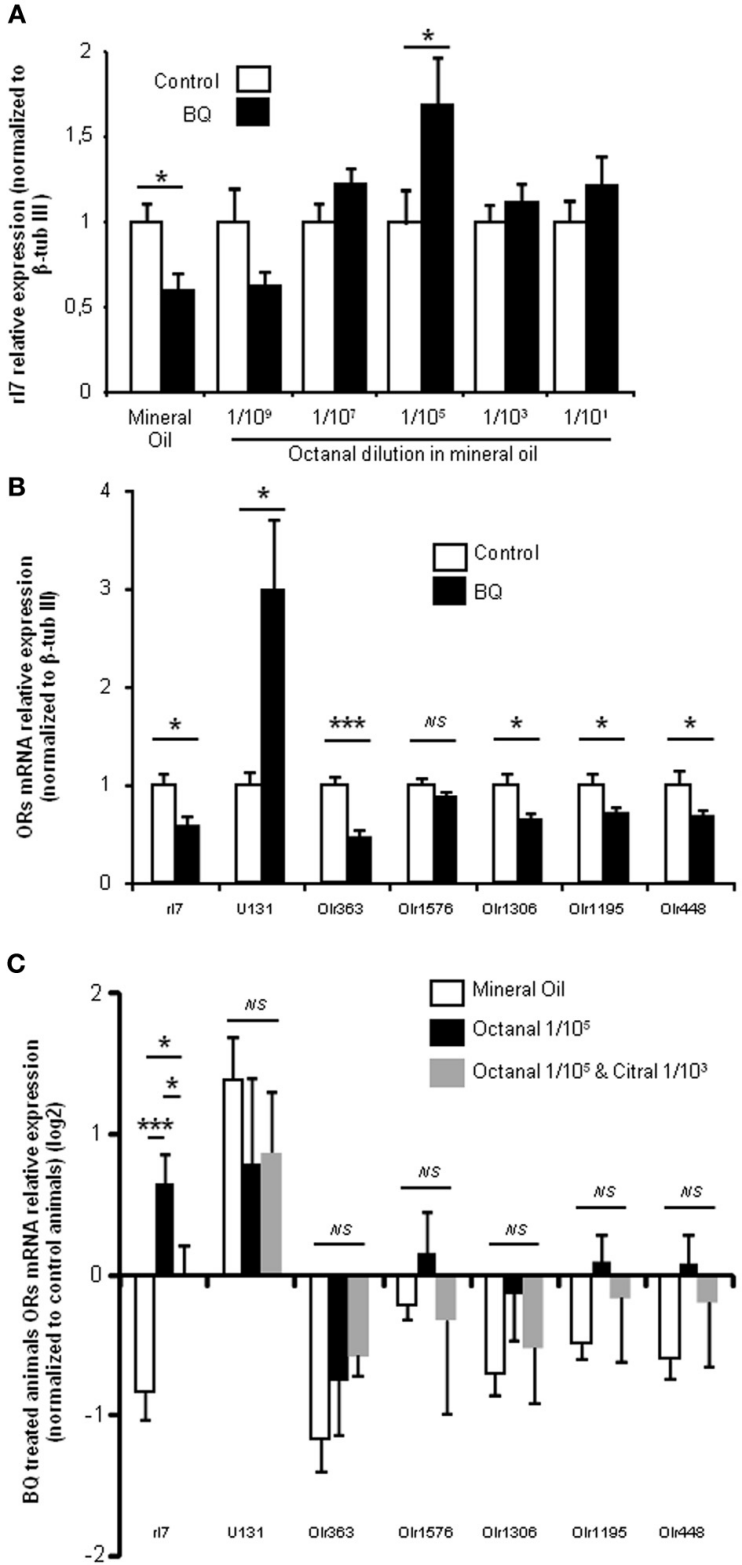
**BQ treatment globally decreases OR transcripts in the OE, and rI7 activation specifically enhances its expression. (A)** qPCR analysis of rI7 expression in the olfactory mucosa of rats treated with BQ or vehicle while odorized with a range of octanal dilutions. **(B)** OR expression analysis for animals treated with BQ and control littermates in presence of mineral oil. **(C)** Effect on OR expression of different odorization during BQ treatment. Values are normalized to control animals and expressed on log_2_ scale. (*n* = 6; ^*^P < 0.05; ^***^P < 0.001).

In order to test the specificity of the odorization on rI7-expressing OSN population, we measured its evolution for various concentrations of octanal. As the manual counting of OSNs after WISH was time-consuming, we indirectly quantified the rI7-OSN population by qPCR. An extensive previous study has demonstrated a good correlation between the number of OSNs expressing a specific OR and its mRNA level in the olfactory mucosa (Khan et al., 2011). In order to verify that it holds true as well in the present case, we first quantified the level of rI7 mRNA after treatment with endothelin receptor antagonists. It was significantly decreased compared to control littermates (0.6 ± 0.09 vs. 1 ± 0.12, respectively; Figure 4A). Odorization with octanal significantly reversed this decrease, a result consistent with the WISH experiments. This effect was dose dependent, as it was ineffective at low concentration (1/10^9^ v/v octanal in mineral oil) and most effective at 1/10^5^ (1.69 ± 0.28 vs. 1 ± 0.2, respectively). In order to further test the specificity of the octanal effect on rI7-expressing OSN population in a pro-apoptotic context, we evaluated the modulation of other ORs mRNA level in the same conditions. To our knowledge; apart from rI7, U131 (Olr748), activated by low chain fatty acids (Murrell and Hunter, 1999; Glatz and Bailey, 2010), is the only deorphanized OR in rats. We quantified the level of mRNA coding for U131 and 5 other putative ORs genes (Olr363, Olr1576, Olr1306, Olr1195, and Olr448, accession numbers in Additional file 2) sharing high homology (92–94%) with mouse deorphanized ORs (putative ligands for those ORs in Additional file 2). Among those 6 ORs, the mRNA expression levels of 4 were statistically decreased by the treatment with endothelin receptor antagonists; one was not statistically affected and another (U131) significantly increased (Figure 4B). We quantified the change induced by BQ treatment on OR mRNA expression with different odorant environments. The effect of BQ treatment was affected by octanal odorization only for rI7 (0.69 ± 0.28 vs. −0.8 ± 0.09 for octanal and control odorization, respectively; Figure 4C). Finally, co-odorizaton with citral, a known antagonist of rI7 (Araneda et al., 2000), was sufficient to limit the effect of octanal odorization (0.15 ± 0.15 vs. 0.69 ± 0.28, respectively). The modulation by citral was effective only on rI7 among all ORs tested (Figure 4C).

## Discussion

We had previously found that endothelin acts as an anti-apoptotic factor for OE cells *in vitro* (Laziz et al., 2011). In the present work, we explored the consequences of an early intranasal treatment with endothelin receptor antagonists. This treatment doubled the basal level of apoptosis in the upper layers of the OE containing sustentacular cells and OSNs. This increase of apoptosis level was consistent with the lower EOG signal amplitudes recorded. A similar variation of EOG amplitude has been observed after unilateral naris occlusion where cellular dynamics is also altered (Waggener and Coppola, 2007). Our results showed that endothelin acts as an anti-apoptotic factor *in vivo* and that the treatment with its receptor antagonists increases the OSN death rate. Surprisingly, despite this higher OSN death rate, animals performed better in an odor based orientation task. As substances applied to the OE could then spread to the whole body, this result may be due to non-sensory effects. For instance, the increase of olfactory performances on the treated group may be due to a change of motivation to find their mother if the treated pups felt sick. However, it seems unlikely because their velocity was not different from their control littermates and their body weight was not altered (18.2 ± 0.43 vs. 18.1 ± 0.49 g, *n* = 12). To explain this discrepancy, we thus hypothesized that the OE of the treated group would be tuned faster to odors already present in the environment.

### Improved olfactory performance after pro-apoptotic treatment in the OE

The pioneer works of Hubel and Wiesel or Hollyday have clearly demonstrated that the nervous system is built according to external and internal signals (Wiesel and Hubel, 1963; Hollyday et al., 1977). Although controversial, numerous studies have pointed out that OSN population exhibits plasticity and adapts to environmental odorants, thanks to its regenerating ability. In order to demonstrate that this is also the case with the treatment applied here, we needed to quantify a population of OSNs expressing a deorphanized OR. We thus focused on the modulation of rI7-expressing OSNs. The treatment diminished their number on turbinate IV where it is mostly localized in 10 days old pups (Vassar et al., 1993). We did not observe any change on other turbinates but it may be simply due to the much lower number of rI7-expressing OSN present there. This first result was consistent with the increased apoptosis level and decreased EOG signals. In presence of octanal this effect was reversed, indicating that the odorization protocol improved the survival of rI7 OSNs. Does this modulation hold true for other ORs? In rat, in addition to rI7, only U131 has been deorphanized. We therefore investigated the effect of the treatment on its expression, along with 5 other putative OR transcripts sharing high homology with deorphanized mouse ORs. A decreased expression was observed for all with the exception of Olr1576 and U131. This result reflects BQ treatment effectiveness in raising OE apoptosis levels. Olr1576 expression was not altered by BQ treatment while U131 transcripts were over expressed in treated animals. This result is only consistent with our hypothesis if the ligands of Olr1576 and U131 are present among the pup's environment odorants. While Olr1576 ligand is unknown, U131 is activated by short chain fatty acids (Murrell and Hunter, 1999; Glatz and Bailey, 2010). As those fatty acids are an important constituent of rat milk (Grigor and Warren, 1980), they are indeed present in the pup's environment. Overall, the combined results of maternal orientation behavior, and modulation of U131 and rI7 expression strengthen the suggestion that an increased apoptosis context enhances the effect of activity-driven survival.

How could this modulation explain our behavior assay results? In rodents, the mother odorant signature is a blend that can be learned by pups (Logan et al., 2012). If the OSN population sensitive to this blend of odorants is following the same evolution as rI7 and U131, then the ratio of OSNs sensitive to this blend will be higher in treated pups. Accordingly the signal to noise ratio generated by OSNs sensitive to dam's nest odorants and transmitted to the olfactory bulb will be higher in treated animals. Such increase in signal to noise ratio is critical to improve the discrimination capacity of the olfactory system (Laurent, 1999). In our behavior test, we used dam's feces and sawdust as attractants for pups which reflect the dam's nest blend. Although part of those modulations could be based on a change of OSN sensitivity to odorants present in the environment, the improved ratio of OSNs sensitive to this blend could explain their improved olfactory performance in our odor-based orientation task.

### Modulation of OSN populations is dependent of odorant environment

We explored the effect of increasing concentrations of octanal on rI7-expressing population by quantifying the level of rI7 mRNA in the OE. The change in mRNA level between control and treated pups was larger than the change in OSN number evaluated by WISH. Indeed, in presence of mineral oil only, while the number of rI7 OSN was diminished by ~27% on turbinates, the level of mRNA coding for rI7 was almost halved. Furthermore, while the odorization with 1/10^5^ octanal rescued the number of rI7 OSN back to control value when evaluated using WISH, the rI7 mRNA level was significantly increased compared to control. This discrepancy could be explained by the partial evaluation of OSN population by WISH, limited to turbinate's surface, while the mRNA quantified by qPCR are extracted from the whole turbinates OE. To our knowledge, there is no published data on the impact of different concentrations of the same odorant on OSN population. First, the effect of odorization was dose dependent. Furthermore, the increase of rI7 mRNA level by octanal exposure was reduced by co-odorization with citral, known to antagonize rI7 (Araneda et al., 2000). Finally, the odorization and its antagonism were most effective on rI7 among all ORs tested. These results are thus consistent with a specific effect of odorization with octanal on rI7-expressing OSNs population. They also indicate that the activity-driven survival is most effective with a low concentration of odorant, which could be related to the lower activity of ORs for high concentrations of its ligand (Duchamp-Viret et al., 1999; Wade et al., 2011). Endothelin receptors are GPCR known to activate the JNK/c-Jun pathway (Nelson et al., 2003). Interestingly, ORs have recently been linked to the MAPK pathway including JNK (Benbernou et al., 2013). It raises the question whether a potential cross talk between survival factor receptors and ORs exists. When impairing the endothelin receptors signaling, the level of JNK pathway activation could decrease, allowing ORs signaling through JNK to be more effective leading to enhanced activity-driven survival of OSNs.

### Faster tuning of the OE to environment odorants in a pro-apoptotic context

In the pro-apoptotic context induced by intranasal delivery of endothelin receptor antagonists, the odorization was effective in reversing the decrease of OSNs number and in improving olfactory performance for environmental odorants after 10 days of exposition. Meanwhile, the rI7 OSN population did not change in the non-treated group which is consistent with a previous study using a similar protocol (Kerr and Belluscio, 2006). Thus, in a physiological context, the olfactory system may be tuned more slowly to environmental odorants by modulation of OSNs survival. This concurs with the findings from a recent study focused on H2BE, an OSN specific histone, which turned out to be essential for their activity dependent survival (Santoro and Dulac, 2012). The authors explored the olfactory performance of mice with deficient H2BE, i.e., limited modulation of OSN population by environmental odorants. Such mice did not differ from WT mouse in their olfactory performances before 3 weeks of odorization. As the OM is permanently regenerating, OSNs have a limited lifespan of approximately 1 month. If activity-dependent survival of OSN impacts the OSN population in 1 week as we observed when apoptosis level is increased, it would lead to a quick tuning of the olfactory system to odorants present in the environment. On the other hand, this quick tuning may impair the ability to detect novel odorants as the never stimulated OSN may disappear too fast from the OM. Thus, a slower modulation of OSN survival driven by their activity (around 3 weeks as observed by Santoro and Dulac in their 2012 study) may benefit the animal by improving the detection of odorants frequently encountered in its environment, without impairing the detection of novel odorants that may be crucial for the survival of the individual.

In an artificial pro-apoptotic context, we altered OSN population dynamics, accelerating the tuning of the olfactory system to the environment through an enhancement of activity-driven OSN survival. This result needs to be extended on mice where more ORs are deorphanized. This may explain why the literature on the importance of activity-driven mechanisms has been so divergent for OSNs. Indeed, this slow modulation of cellular dynamics would appear only in an altered physiological state, explaining why it is ruled out in a physiological context (Kerr and Belluscio, 2006). The literature controversy on this subject may also partly be explained by the odorization protocol used. If our results on rI7-expressing OSN holds true for other ORs, a low concentration of odorant leads to greater effects and such low concentrations have not been used in previous studies of activity-dependent survival of OSNs.

## Materials and methods

### Chemicals

BQ_123_ and BQ_788_ (ET_A_ and ET_B_ antagonists, respectively), were purchased from Sigma Aldrich (Saint-Quentin Fallavier, France; Catalog numbers B150 and B157, respectively).

### Animals

All animals were housed at our local animal care facilities (UEIERP; Jouy-en-Josas; France) in 12 h light, 12 h dark cycles with free access to food and water. Experiments were performed on litters of 12 newborn male Wistar rats with one mother. Pups were treated from birth to 10 days; twice a day (at 9 a.m. and 6 p.m.) with 3.5 (at birth) to 8 μL (increasing the volume by 0.5 μL every day) per nostril of either a mixture of BQ_123_ and BQ_788_ at 10^−5^ M in PBS (Mixture of endothelin receptor antagonists; BQ) or PBS with 10^−4^% of NaOH (pH = 7.6; Vehicle taking into account the initial dilution of BQ_788_ on 0.01% NaOH). Their lactating mother was concurrently odorized by spraying her nipples with 200 μL of mineral oil or various concentrations of odorant diluted in mineral oil. All animal experiments were approved by the local ethics committee (COMETHEA; Avis 12/063) and conducted in accordance with the European Communities Council Directive of November 24, 1986 (86/609/EEC). Except otherwise specified in the following sections, rats were sacrificed at the beginning of the light phase (09:00–10:00) by decapitation.

### Apoptosis quantification in the OE by tunel

To determine the level of apoptosis in the OE after treatment with either BQ or vehicle, we quantified the level of TUNEL staining from tissue sections of 10 days old pups as described previously (Laziz et al., 2011). We used 10 pups for each condition coming from 2 separated experiments. The nasal septum and turbinates were removed as a block and post-fixed overnight at 4°C in 4% paraformaldehyde PBS. Blocks were cryoprotected with sucrose (30%) and cryo-sectioned sagitally (14 μm thick). Sections were kept frozen at −80°C until use. We performed terminal deoxynucleotidyltransferase-mediated dUTP nick end-labeling following provider instruction (DeadEndTM Fluorometric TUNEL system, Promega, Charbonnières-les-Bains, France) on four transversal sections per rat, spread regularly through the OM. Sections were finally stained with 2 μg/mL Hoechst 33342 for 10 min and mounted in Vectashield after extensive wash in PBS. For all sections, we took four images located dorso-medially at the base of the septum corresponding to zone 1 as defined previously (Ressler et al., 1993). Images were taken blindly of the treatment at x100 magnification. Images were quantified using ImageJ (Rasband, W.S., ImageJ, U. S. National Institutes of Health, Bethesda, Maryland, USA, http://imagej.nih.gov/ij/, 1997–2012) to threshold specific TUNEL staining as described previously (Laziz et al., 2011). The area of the OE was measured from the Hoechst staining allowing us to quantify the percentage of TUNEL staining area in the OE (See an example of quantification in Additional file 4). The same threshold was applied for all images arising from the same experiment performed on the same litter. All results were expressed as a relative value of the control group treated with vehicle only.

### Electroolfactogram recordings (EOG)

To evaluate the global responses of OSNs after treatment with either BQ or vehicle, EOG recordings were made from the olfactory mucosa in an opened nasal cavity configuration as described earlier (Negroni et al., 2012). We used 12 pups for each condition coming from 2 separated experiments. Pups were sacrificed by decapitation during light phase (09:00–18:00) to allow continuous recordings during the working day but we took care to alternate them according to their treatment to limit any circadian bias. The hemi-head was placed in a recording chamber under an upright Olympus SZ51 stereo microscope (Olympus, Rungis, FRANCE) equipped with a low magnification objective (0.8–4×) and two MX-160 micromanipulators (Siskiyou, Inc., Grants Pass, OR, USA). The odor stimulation device was modified from Scott and Brierley (Scott and Brierley, 1999). The hemi-head was kept under a constant flow of humidified filtered air (~1000 ml/min) delivered through a 9 mm glass tube. This tube was positioned 2 cm from the epithelial surface. Odor stimulations were performed by blowing air puffs (200 ms, 200 ml/min) through an exchangeable Pasteur pipette enclosed in the glass tube containing a filter paper impregnated with 20 μL of odorant. We used a mixture of 12 odorants (equimolar mixture of anisole, citral, heptanal, isoamyl acetate, Lyral, lilial, octanol, 1-4-cineole, isomenthone, limonene, L-carvone and pyridine) diluted to a final concentration of 10^−3^ M; isoamyl acetate ranging from 1:10000 to 1:10 and citral from 1:1000 to 1:10. Odorants were diluted in mineral oil (Sigma Aldrich, Saint-Quentin Fallavier, France, catalog number: M3516). To prevent variable accumulations of the odorant in the pipette, an air flush was applied to the Pasteur pipette before it was placed in the glass tube.

EOG voltage signals were recorded using an XtraCell 2 channels amplifier (DIPSI, Chatillon, FRANCE) used in a DC current-clamp configuration (*I* = 0), low-pass bessel filtered at 1 KHz and digitized at a rate of 2 kHz using an Digidata 1322a A/D converter (Axon Instruments, Molecular Devices, Union City, CA, USA) interfaced to a Pentium PC and Pclamp 9.2 software (Axon Instruments). A reference Ag/AgCl electrode was placed on the frontal bone overlaying the olfactory bulb. Recordings were made with glass micropipettes of 4–5 MΩ filled with a saline solution. EOG were recorded from the center of turbinates IIb and III. These positions gave robust, reproducible and long-lasting EOG recordings ranging from 7 to 14 mV when stimulated with the mixture of odorants at 10^−3^ M. Odorant-free air stimulation (with mineral oil), always produced signals around 1 mV amplitude. Analyses were performed using Clampfit 9.2 (Axon Instruments) in order to measure the peak amplitude.

### Olfactory behavior test

In order to evaluate olfactory performances of pups based on videotracking, we designed a new behavioral test based on the orientation of the young rats toward their maternal odor. Tests were conducted between 10:00 and 11:00 a.m. on two litters of 12 male Wistar rats of 10 days. Pups were separated from their mother 45 min prior testing and marked with an odorless marker for video-tracking purpose. Spots were made at the center of the head and at the center of the body (Figure 3A). Pups were then introduced individually in the center of a closed custom-made box (L × W × H: 29.7 × 18.7 × 5.5 cm^3^), with a continuous airflow incoming from two opposite compartments expelled by a small fan at the base of the box (~30 L.min^−1^; i.e., ~10 box volume.min^−1^). One compartment contained sawdust mixed with feces collected from the dam while the other only contained fresh sawdust. Videotaping was performed at 15 frames per second for 1 min or less if the animal tried to enter one of the compartments indicating a choice between the two odor sources. Experimental pup order was alternated according to their treatment while keeping exactly the same odor sources.

Videos were analyzed to track both spots (head and body) using Kinovea freeware (http://www.kinovea.org/). We used the coordinates to calculate two unit vectors, one represented by the body dot to head dot (animal orientation vector) and one by the body dot to mother litter compartment (perfect orientation vector) (Figure 3B). For each frame, we calculated the scalar product of those vectors. A value of 1 indicates that both are aligned and thus the animal is perfectly orientated toward its mother smell while a scalar of −1 indicates an opposite direction. If the animal chooses a compartment before the 1 min testing limit, the scalar value was assigned to 1 or −1 for the remaining frames according to the chosen compartment. The scalar values are then averaged for all frames to calculate a score of odor based orientation for each pups. We also monitored their average velocity.

### Whole mount *in situ* hybridization (WISH)

In order to quantify variations of rI7 OSN population we performed whole-mount *in situ* hybridization following a published protocol with modifications (Vassar et al., 1993). For this purpose rI7 ORF has been cloned into pGEM®-T (Promega) and used as template for *in vitro* transcription to generate DIG-labeled RNA sense and antisense probes (DIG RNA Labelling Kit (SP6/T7), ROCHE). Blocks from rat hemi-heads containing olfactory mucosa were fixed overnight in 4% paraformaldehyde PBS and then dehydrated in sucrose (30%, 24 h) and in successive methanol baths (48 h). Tissues were rehydrated before permeabilization using proteinase K (10 μg/ml) for 10 min at 37°C, washed twice in PBS/glycine (2 mg/mL) and incubated in post-fixation solution (4% PFA and 0.2% glutaraldehyde) for 20 min. The digoxygenin-labeled probes (0.1 μg/μl) were denaturated beforehand at 85°C for 5 min then added in the hybridization mix (50% formamide; 5× SSC; 1% blocking reagent; 0.1% tween; 0.5% CHAPS and 5 mM EDTA) and hybridized overnight at 65°C. Blocks were then sequentially washed in 50% formamide/1% SDS/5× SSC (2 × 30 min at 65°C) and 50% formamide/1% SDS/2× SSC (20 min at 65°C). Immuno-detection was performed using the DIG Nucleic Acid Detection kit (Roche) following the supplier recommendations. Hybridized probes were revealed by overnight incubation at room temperature in the dark with NBT-BCIP as substrate in detection buffer. Reaction was stopped by rinsing sections in PBS. Blocks were briefly washed in methanol (1 min) in order to decrease background coloration. Images of each turbinate were taken by an operator blinded to the treatment under X40 magnification on a DMBR Leica microscope equipped with an Olympus DP-50 CCD camera using CellF software (Olympus Soft Imaging Solutions GmbH, OSIS, Münster, Germany). rI7 OSN were counted using ImageJ software (Rasband, W.S., ImageJ, U. S. National Institutes of Health, Bethesda, Maryland, USA, http://imagej.nih.gov/ij/, 1997–2012).

### qPCR

Total RNA was extracted using the Trizol method from frozen olfactory epithelia. OligodT first strand cDNA were synthesized from 2 μg total RNA by the Superscript II reverse transcriptase (Invitrogen) following the manufacturer recommendations then treated with DNase I. For quantitative PCR, 5 μl of 125 fold diluted cDNA templates were added to the 15 μ l-reaction mixture containing 300 nM primers (sequences in Additional file 2, melting curves in Additional file 3) and SYBR Green GoTaq® qPCR Master Mix (Promega, Charbonnieres, France). The PCR was performed on a Mastercycler® ep realplex (Eppendorf) during 40 amplification cycles consisting in 45 s at 94°C, 45 s at 60°C and 45 s at 72°C. Quantification was achieved using the ΔΔCt method. mRNA expression was normalized to the expression level of the OSN specific β-tubulin III (Khan et al., 2011) and efficiency corrective factor was applied for each primer pair.

### Statistical analysis

Data are expressed as mean ±standard error of the mean (s.e.m). The Student's *t*-test were used to determine statistical significance of differences between groups, except for WISH experiments statistically analyzed with a factorial ANOVA followed by LSD *post-hoc* test and qPCR results for odorized animals analyzed using a One-Way ANOVA followed by Newman-Keuls multiple comparison *post-hoc* test. A probability value of at least *P* < 0.05 was used as an indication of significant differences.

## Author contributions

Nicolas Meunier designed and performed experiments, analyzed data and wrote the paper; Adrien François, Iman Laziz, Stéphanie Rimbaud designed and performed experiments and analyzed data, Denise Grebert, Didier Durieux performed experiments; Adrien François and Edith Pajot-Augy edited the manuscript.

### Conflict of interest statement

The authors declare that the research was conducted in the absence of any commercial or financial relationships that could be construed as a potential conflict of interest.

## Supplementary material

Additional file 1 **Behavioral assay movie Movie corresponding to Figure 2B_1_ pathway**. The tracking of the two dots used for scalar quantification is incrusted within the video.

Additional file 2 **List of primers used for qPCR analyses**.

Additional file 3 **Melting curves of primer pairs used for qPCR analyses**.

Additional file 4 **Illustration of TUNEL area quantification on images presented in Figure 1**.
