# Supplementary material for: Early survival factor deprivation in the olfactory epithelium enhances activity-driven survival
Source: Front Cell Neurosci. 2013 Dec 24;7:271. doi: 10.3389/fncel.2013.00271 (PMC3870945; doi:10.3389/fncel.2013.00271)
Supplement: Additional file 2 — List of primers used for qPCR analyses. [file DataSheet1.PDF]

Gene *Accession number*	Ligand	Sense primer	Antisense primer	Amplicon size (bp)
**Olr226 (rl7)** *NM_031710.1*	**Octanal**	5' - CTCCCACCTCACTGTTGTGAT	5' - GCGTAGAGTACAGAGACCAGCTT	111
**Olr748 (U131)** *NM_001000365.1*	**Heptanoic acid**	5' - TGGCAAGTAAAATTTGGTGCTA	5' - CCAGTCAGAATGAACTCAGGAA	104
**Olr363** *NM_001000754.1*	**Lyral/Lilial** *Putative*	5' - AGGCTGCCTGTCACAAAT	5' - TGTAATGAAGGGGGAAGCAG	113
**Olr1576** *NM_001000499.1*	**Lyral** *Putative*	5' - TGGCAGGTTGTGCAACCCAAAT	5' - GAAGGGCAAATGGAACATGGCT	224
**Olr1306** *NM_001000465.1*	**Eugenol** *Putative*	5' - ATTTGTCACCCTCTGCGGTACT	5' - ACCTGAGGCCAATGGCAAGATA	195
**Olr1195** *NM_001001081.1*	**Acetophenone** *Putative*	5' - TCTTCCTCAGCAACCTGTCACT	5' - ACATAGCGGTCATAGGCCATCA	190
**Olr448** *NM_001000287.1*	**2-heptanone** *Putative*	5' - TGGGGAATCTGGGAATGATCGT	5' - AGCTGGATAGCACAACCCAAGA	184
**β-tub III** *NM_139254.2*	-	5' - TGAGGCCTCCTCTCACAAGT	5' - GGCCTGAATAGGTGTCCAAA	105

Additional file 2 | List of primers used for qPCR analyses.

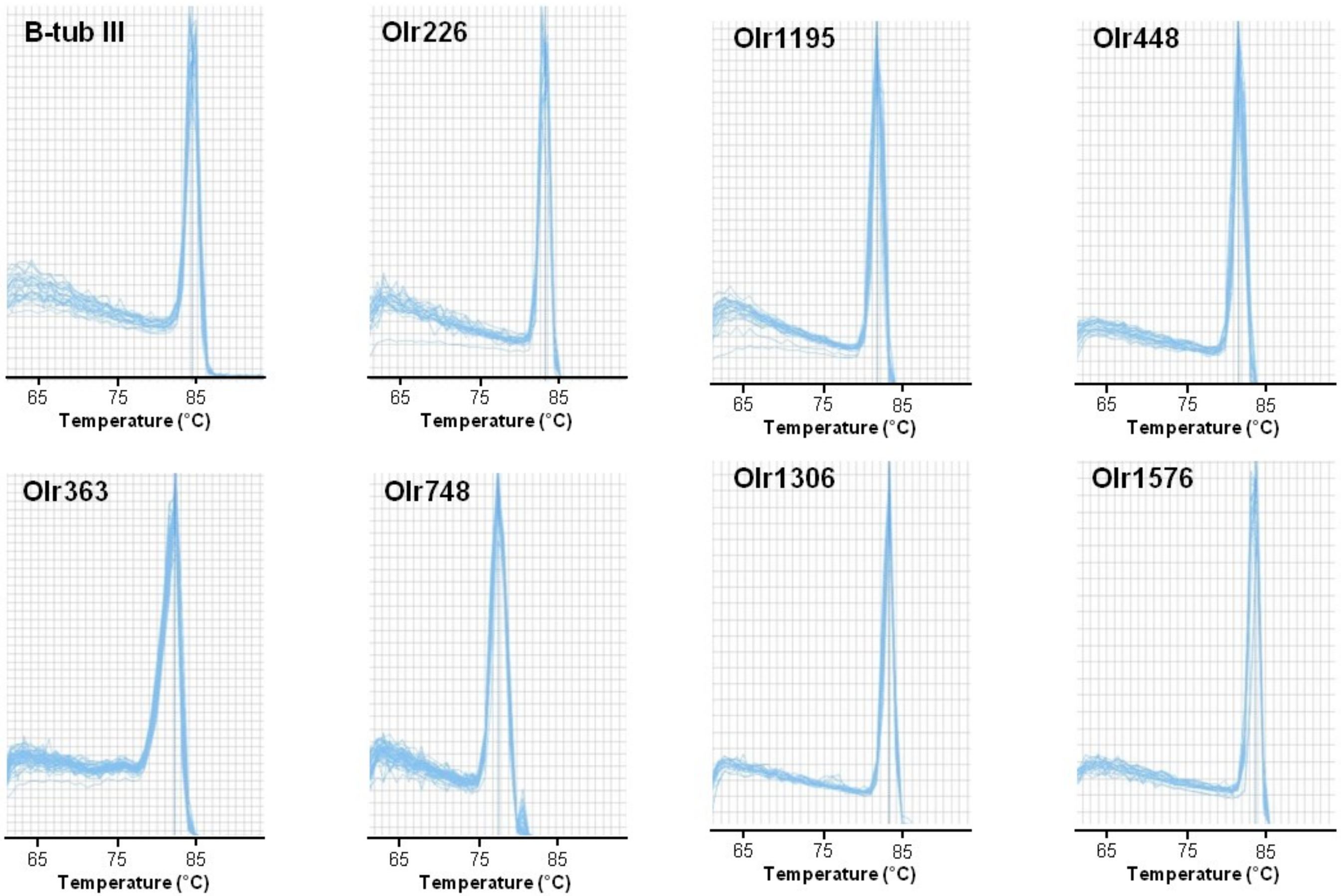

Additional file 3 | Melting curves of primer pairs used for qPCR analyses.

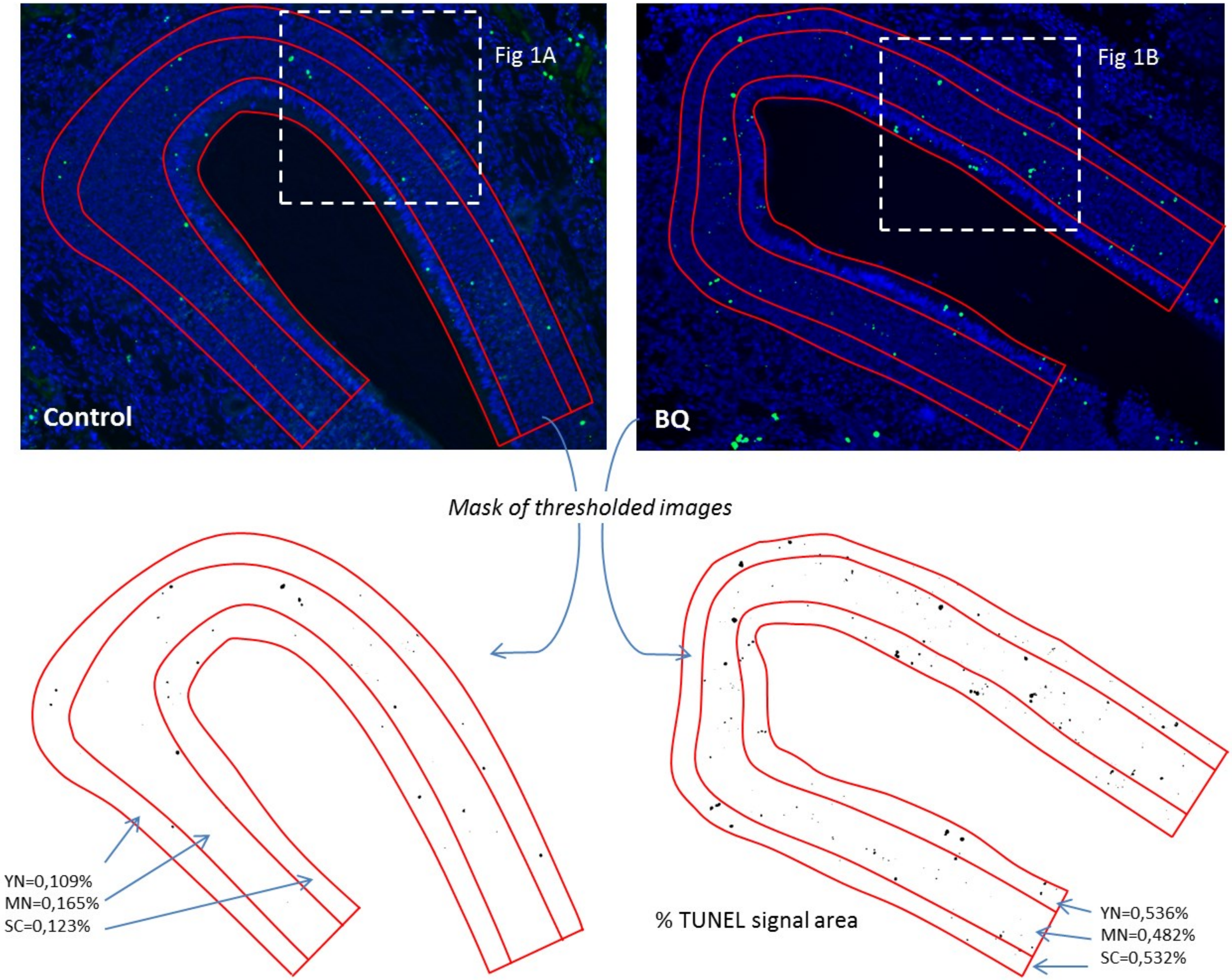

Additional file 4 | Illustration of TUNEL area quantification on images presented in Figure 1.
